# The Clinical Application of Anti-CCP in Rheumatoid Arthritis and Other Rheumatic Diseases

**Published:** 2007-05-03

**Authors:** CT Chou, HT Liao, CH Chen, WS Chen, HP Wang, KY Su

**Affiliations:** 1 Division of Allergy-Immunology-Rheumatology, Veterans General Hospital; 2 Division of Allergy-Immunology-Rheumatology, Wan Fang Hospital, Taipei, Taiwan

**Keywords:** Anti-CCP antibody, rheumatoid factor, rheumatoid arthritis, HLA-Class II genes, smoking

## Abstract

Rheumatoid arthritis (RA) is a common rheumatic disease in Caucasians and in other ethnic groups. Diagnosis is mainly based on clinical features. Before 1998, the only serological laboratory test that could contribute to the diagnosis was that for rheumatoid factor (RF). The disease activity markers for the evaluation of clinical symptoms or treatment outcome were the erythrocyte sedimentation rate (ESR) and C-reactive protein (CRP). As a matter of fact, the diagnosis of early RA is quite impossible, as the clinical criteria are insufficient at the beginning stage of the disease. In 1998, Schelleken reported that a high percentage of RA patients had a specific antibody that could interact with a synthetic peptide which contained the amino acid citrulline. The high specificity (98%) for RA of this new serological marker, anti-cyclic citrullinated antibody (anti-CCP antibody), can be detected early in RA, before the typical clinical features appear. The presence or absence of this antibody can easily distinguish other rheumatic diseases from RA. Additionally, the titer of anti-CCP can be used to predict the prognosis and treatment outcome after DMARDs or biological therapy. Therefore, with improvement of sensitivity, the anti-CCP antibody will be widely used as a routine laboratory test in the clinical practice for RA.

## Rheumatoid Factor and Anti-CCP Antibody in Rheumatoid Arthritis

Rheumatoid arthritis (RA) is a systemic autoimmune disease of unknown origin, characterized by chronic joint inflammation that may later develop into joint destruction, as well as functional limitation (1, 2). So far, the diagnosis has mainly depended on clinical manifestations. The laboratory test which may contribute to the diagnosis of RA is that for rheumatoid factor (3–6). However, the positive rate is approximately 70%. Since many rheumatic or immune diseases, including systemic lupus erythematosus (SLE), Sjogren’s syndrome (SS), primary cryoglobulinemia, and viral infection or tumor may develop positive RF, the specificity of RF in RA is apparently lower (4,7–9). Therefore, it is necessary to search for other laboratory diagnostic markers with high sensitivity and high specificity.

Since 1960, many investigators used indirect immunoflurorescence and enzyme-linked immunosorbent tests to detect serologic antibodies in RA patients (10–15). These consisted of anti-perinuclear antibody (APF), anti-keratin antibody (AKA), anti-filaggrin antibody (AF), anti-Sa, anti-RA 33, and others. Although the specificity was higher (88%–99%) in some of those tests, the overall sensitivity (36%–59%) was lower and thereby limited their use as a routine laboratory test in RA.

Schelleken in 1988 reported that 76% of RA patients had a specific antibody which could interact with a synthetic peptide which contained the amino acid citrulline (16). The arginine of the original substrate from APF or AKA can be converted through a PAD (peptide arginine deaminase) enzyme to “citrulline”, which can be easily detected by anti-CCP antibody (anti-cyclic citrullinated antibody) (17–20). This modification actually improves the specificity up to 98%. Sensitivity recently increased to nearly 80% after we used the 2nd generation anti-CCP enzyme-linked immunosorbent test (ELISA) (21–23).

In the past 5–6 years, many studies have focused on the value of the clinical application of anti-CCP antibody in rheumatoid arthritis and other rheumatic diseases (21–25). The high specificity (98%) of anti-CCP in patients with RA can exclude other rheumatic or immune diseases in patients with positive anti-CCP (26–35). In addition, the anti-CCP antibody test may help us detect or recognize RA earlier (6, 36–43). In patients with RA, recent studies also demonstrated that high anti-CCP antibody had a poor radiological outcome (24, 25).

### Anti-CCP in other rheumatic diseases

Many rheumatic or immune diseases can present the clinical symptoms of polyarticular, symmetrical arthritis and positive RF, which mimic RA or fulfill the diagnosis of RA. These consist of SLE, SS, psoriatic arthritis (PSA) with polyarticular involvement, HIV-related arthropathy, polymyalgia rheumatica (PMR), and even undifferentiated arthritis or palindromic rheumatism (PR) (26–37).

Anti-CCP antibody can be detected in a small percentage of patients with either rheumatic disease or immune disease (Table 1). One study measured the anti-CCP antibody in 126 patients with PSA. Only 7 out of 126 (5.6%) patients were positive for anti-CCP (27). However, the presence of anti-CCP antibodies in PSA was significantly associated with the HLA-DR1 shared epitope (p < 0.005), and even erosive disease (p < 0.05) or a number of swollen joints (p < 0.02) (27). Interestingly, Bockelmann found 11 of 62 (17.7%) psoriasis patients with PSA had a positive anti-CCP antibody, which was significantly increased compared to the control group (4.1%, p < 0.01) (33). Gottenberg studied 134 patients with primary SS, and 10 of the 134 patients (7.5%) exhibited positive anti-CCP (30). Whether the real prevalence of anti-CCP in primary SS or the positive anti-CCP in primary SS patients renders them prone to developing RA requires long-term follow-up.

Palidromic rheumatism is a common rheumatic disease, characterized by recurrent self-limited arthritis. More than 50% of PR may evolve into RA years later. Salvador et al. demonstrated 18 of 32 (56.3%) patients with pure PR had serologically positive anti-CCP antibody. The clinical syndrome can be considered as an abortive form of RA (44). Russell, in his early study, showed that 34% of cases with PR after years of follow-up (mean duration 6 years) had progressed to RA (45). Recently, the same group demonstrated 29 of 61 PR patients (nearly 50%) had progressed to RA. Among those 29 cases, 83% had had anti-CCP antibodies in their baseline sera (36). They concluded that anti-CCP antibodies were better than RF in predicting outcome.

RF can be detectable in cases with hepatitis C infection, with a prevalence ranging from 30% to 60% (34, 46–48). In contrast to hepatitis C, the prevalence of hepatitis B virus (HBV) carriers in ethnic Chinese populations, including Taiwanese, was higher than in Caucasians. Therefore, the positive rheumatoid factor in Chinese with polyarthritis easily leads to the misdiagnosis of RA in HCV or HBV carriers. The lower prevalence of anti-CCP antibody in HCV-infected patients with arthralgia (5.7%) reported by Sene et al. (34) and 0% by Bombardieri (31) suggests anti-CCP antibodies are reliable markers to distinguish HCV-related arthropathy or Sjogren’s syndrome from RA (30, 31, 34, 46, 47).

PMR is a rheumatic disease that mainly affects elderly people. The persistent shoulder pain with morning stiffness and remarkable elevation of ESR or acute phase reactant makes it difficult to distinguish PMR from elderly onset RA (EORA) with initial shoulder joint involvement (49). A recent study by Lopez-Hoyos showed that 75% of EORA patients had anti-CCP antibodies, whereas none of the PMR patients was positive for those antibodies (49). Therefore, the positive RF and anti-CCP antibodies can exclude the possibility of PMR early on.

### The impact of rheumatoid factor and anti-CCP on extraarticular manifestations of RA

The extraarticular manifestations of RA consist of rheumatoid nodules, interstitial lung disease or pleuritis, vasculitis, amyloidosis, scleritis, mononeuritis, and others. The positive correlation between RF and entraarticular manifestations was reported by De Rycke (50). However, Korkmaz did not find a relationship between RF and extraarticular manifestations (51).

Although anti-CCP antibodies are associated with the severity of RA and erosion, both Rycke and Korkmaz could not demonstrate a positive correlation between anti-CCP antibodies and extra-articular manifestations (50, 51). Many factors may affect the negative results, including patient sample sizes, disease duration and treatment (50, 51). Long-term follow-up of RA patients is required to understand the association between anti-CCP and extraarticular manifestations.

### What is the relationship between the genetic and environmental factors of RA and anti-CCP antibody?

RA has long been recognized as an autoimmune disease which is genetically determined. HLA-Class II alleles are the most important genetic marker that increases the risk of developing RA (52). The shared epitope (SE) of HLA-Class II alleles indicates they share the conserved amino acid sequence, and constitute a part of the antigen-binding site (53). These SE alleles are located in the third hypervariable region of the HLA-DRB1 molecules (QKRAA, QRRAA or RRRAA). Many SE alleles have been studied, including DRB1, 0101, 0102, 0104, 0401, 0404, 0405, and 0408, and among them, 0101, 0401 and 0404 were highly associated with RA in Caucasians (54–57). To explore the association among SE, anti-CCP and RA, many investigators found that HLA-DRB1 alleles encoding the SE were only associated with RA in the presence of anti-CCP antibodies, and were not associated with anti-CCP negative RA (58–62).

Recently, the North American RA Consortium family cohort and the Study of New Onset RA cohort further investigated 1723 Caucasian RA patients and reconfirmed that HLA-DRB1 SE was strongly associated with anti-CCP (63). In this study, several interesting findings were observed. Unlike anti-CCP antibody, SE was not significantly associated with the presence of RF (63). Additionally, HLA-DR3 alleles were negatively associated with positive anti-CCP and easily found in anti-CCP negative individuals. In cases with DR3 positive RA, the anti-CCP antibody level was lower compared to DR3 negative RA. Verpoort confirmed the finding that HLA-DR3 was associated with anti-CCP negative arthritis, but not with anti-CCP positive arthritis (64).

The interaction between SE alleles and citrullinated peptide antigen may be the pathogenetic mechanism that increases the anti-CCP antibody production in RA (17). However, citrullinated peptide antigen was found not only in RA synovium, but also in other diseases (65). This was proposed as an abnormal humoral response to these citrullinated proteins in RA patients, and requires further identification.

For juvenile RA, HLA-DR4 positive patients with polyarticular onset were more likely to have anti-CCP antibodies than those without HLA-DR4 (OR 5.20, CI 1.30–20.9) (66). Interestingly, the presence of anti-CCP antibodies in 7 patients with psoriatic arthritis (PSA) was also significantly associated with HLA-DRB1 SE (0101, 0401) (p < 0.005) and erosive disease (27). Among these 7 PSA patients, 4 showed a polyarticular pattern. For either JRA or PSA, the frequency of anti-CCP antibody in these patients was rather low (5–10%), and the presence of anti-CCP associated with HLA-Class II SE was mainly noticed in polyarticular-type cases. Up to now, the relationship between the anti-CCP antibody and SE with a polyarticular subtype in either JRA or PSA remains unknown. Follow-up is strongly indicated, particularly for the JRA cases that eventually develop adult RA or other diseases.

The environmental factors in RA are multiple. Among them, smoking is a risk factors for RA, and this is evidenced by the fact that smokers have increased levels of RF (67–69) and are prone to develop RA (70–72). By combining anti-CCP and SE, Linn-Risker et al. concluded that smoking increases the risk for anti-CCP antibodies only in shared epitope-positive patients with RA (58). Swedish investigators demonstrated that both SE and smoking resulted in an increased risk, specifically for RF positive RA (73).

However, it is quite difficult to identify the possible pathogenic pathways through which smoking confers a high risk of developing RA in SE-positive individuals. Whether smoking can increase the proinflammatory cytokines or destroy the tolerance to citrinullated proteins remains unknown and needs further investigation (58).

### Prognositive factors of anti-CCP antibody in rheumatoid arthritis

Many recent studies have shown that anti-CCP antibody can predict the severity of either the clinical or radiological outcome in RA patients (24, 25, 74–80). Forslind showed that anti-CCP positive early RA patients had a higher Larsen score at baseline compared to anti-CCP negative RA patients, and had significant radiological damage after years of follow-up (25). Kastbom reconfirmed that anti-CCP antibody positivity at diagnosis predicted higher disease activity over the following 3 years of recent-onset RA (24). The presence of anti-CCP antibody or anti-perinuclear factor in RA patients, instead of RF, presented an increased total sharp score after 5 years of follow-up (74). When comparing erosive and non-erosive RA patients, the levels of anti-CCP antibodies and ESR were significantly higher in erosive RA patients (75). Aotsuka et al. measured anti-CCP antibody in RA patients during the period 1982–2004 and found that anti-CCP levels tended to fluctuate in parallel with the ESR or CRP level (78).

We recently enrolled 155 ethnic Chinese RA patients and divided them into anti-CCP positive (110) and anti-CCP negative (45) patients. There were no significant differences in the demographic data (age, gender, disease duration and treatment, etc.) between the 2 groups. Rheumatoid factor was significantly higher in the anti-CCP positive group than in the anti-CCP negative group (77.3% vs. 37.8%, p < 0.0001). All the clinical parameters (number of tender joints and swollen joints) and laboratory data (ESR, CRP) were significantly greater in anti-CCP positive than in anti-CCP negative patients (p < 0.05, < 0.01). The DAS score in the anti-CCP positive group was 4.30 (2.03–7.52) and in the anti-CCP negative group was 3.69 (2.03–6.54), which revealed a significant difference between the 2 groups (p = 0.007) (unpublished data). Our results differed from those of the study by van der Helm et al. who showed no significant difference in disease activity and CRP between RA patients with and without anti-CCP antibodies (77). We have to continue following up our RA patients in order to understand whether the presence of anti-CCP in Chinese RA patients can determine the poor clinical and radiological outcome, as in Caucasian patients.

### Can serum anti-CCP antibody level be used as a useful adjunct in assessing drug efficacy?

The 2 most common laboratory tests that we use to evaluate RA disease activity and treatment efficacy are ESR and CRP. In certain RA patients, the clinical manifestations may not be correlated well with ESR and CRP. Searching for other disease activity markers in order to assess clinical improvement after a specific drug therapy is important. After biological therapy, a significant improvement in clinical features, including pain, stiffness and swelling has been noticed. Apart from that, both ESR and CRP can be reduced significantly within 3 months after treatment (81, 82). Several groups have recently studied the RF and anti-CCP levels before and after TNF-alpha inhibitors (83–88).

The positive rate for anti-CCP antibody before infliximab or etanercept treatment ranged from 83% to 90%, and for RF, from 78% to 95%. The higher frequency of anti-CCP or RF was mainly due to the severe RA patients who were enrolled in the study (81, 83–86, 89). Alessandri treated 43 RA patients with infliximab and found the serum titer of anti-CCP and RF decreased significantly after 6 months of treatment (83). In contrast, there was no significant change in ESR and CRP before and after infliximab. Two other studies demonstrated that RF, but not anti-CCP, was significantly reduced after infliximab treatment. (84, 89). Bobbon-Pallaricini investigated the effect of long-term infliximab treatment (up to 78 weeks) on anti-CCP, RF, anti-DNA, etc. The results showed a significant reduction of RF from 128 IU/ul (baseline) to 53 IU/ul (78 weeks). However, anti-CCP antibody significantly decreased at 30 weeks, but returned to baseline thereafter (85). A more interesting finding was reported by Braun-Moscovici, who demonstrated a positive correlation between lower baseline levels of anti-CCP and clinical response to infliximab (86). Our recent study demonstrated findings similar to those of Alles-sandri, except that the biological agent we used was etanercept (81). A significant reduction of serum levels of anti-CCP and RF was found in patients who received etanercept combined with DMARDs, compared to patients with DMARDs alone (p = 0.007 and p = 0.006 respectively). In addition, a positive correlation between anti-CCP antibody titer and variation in disease activity, swollen and tender joint counts, RF and CRP was observed without biological therapy. DMARDs also reduced the anti-CCP or RF level by 25%, but only shorter disease duration (≤12 months) was significantly correlated with a decline in the levels of anti-CCP antibody (90).

In conclusion, short-term biological therapy (<6 weeks) can effectively suppress the serum anti-CCP level in RA patients. The long-term (>1 year) efficacy of TNF-alpha inhibitor for the serum anti-CCP antibody level is not conclusively known now, and requires further study.

## Conclusion

Anti-CCP has become a “key” serologic marker in RA. It can be used (1) as a test for early diagnosis of RA; (2) for the differential diagnosis between RA and other rheumatic or immune diseases; (3) for prediction of prognosis; and (4) for evaluation of treatment outcome.

## Figures and Tables

**Table 1:** The prevalence of anti-CCP in different rheumatic diseases.

Disease	Prevalence (%) of anti-CCP	Author
1. Psoriatic arthritis Control	17.7% (11/62) 4.1% (9/98)	Bockelmann R (Ref. 33)
2. Psoriatic arthritis Control	5.6% (7/126) 0% (0/97)	Korendowych E (Ref. 27)
3. Sjogren’s syndrome	7.5% (10/149)	Gottenberg JE (Ref. 30)
4. Sjogren’s syndrome	0% (0/7)	Sene D (Ref. 34)
5. HCV (+) with arthragia	5.7% (2/35)	Sene D (Ref. 34)
6. Polymyalgia rheumatica	0% (0/49)	Lopez-Hoyos M (Ref. 49)
7. Palidromic rheumatism (pure form)	56.3% (18/32)	Salvador G (Ref. 44)
